# The 4-1BBζ costimulatory domain in chimeric antigen receptors enhances CD8+ T-cell functionality following T-cell receptor stimulation

**DOI:** 10.1186/s12935-023-03171-7

**Published:** 2023-12-18

**Authors:** Gerard J. Chu, Charles G. Bailey, Rajini Nagarajah, Sharon M. Sagnella, Stephen Adelstein, John E. J. Rasko

**Affiliations:** 1 Gene and Stem Cell Therapy Program Centenary Institute, Camperdown, NSW Australia; 2 Department of Clinical Immunology and Allergy, Royal Prince Alfred Hospital, Camperdown, NSW Australia; 3 Faculty of Medicine and Health, University of Sydney, Sydney, NSW Australia; 4 Cancer & Gene Regulation Laboratory Centenary Institute, Camperdown, NSW Australia; 5 Cell & Molecular Therapies, Royal Prince Alfred Hospital, Camperdown, NSW Australia

**Keywords:** CAR, Signaling, Function, 4-1BB, CD28, Cancer, Immunotherapy, Immunology

## Abstract

**Background:**

Chimeric antigen receptor (CAR) T-cells have revolutionized the treatment of CD19- and B-cell maturation antigen-positive haematological malignancies. However, the effect of a CAR construct on the function of T-cells stimulated via their endogenous T-cell receptors (TCRs) has yet to be comprehensively investigated.

**Methods:**

Experiments were performed to systematically assess TCR signalling and function in CAR T-cells using anti-mesothelin human CAR T-cells as a model system. CAR T-cells expressing the CD28 or 4-1BB costimulatory endodomains were manufactured and compared to both untransduced T-cells and CAR T-cells with a non-functional endodomain. These cell products were treated with staphylococcal enterotoxin B to stimulate the TCR, and in vitro functional assays were performed by flow cytometry.

**Results:**

Increased proliferation, CD69 expression and IFNγ production were identified in CD8+ 4-1BBζ CAR T-cells compared to control untransduced CD8+ T-cells. These functional differences were associated with higher levels of phosphorylated ZAP70 after stimulation. In addition, these functional differences were associated with a differing immunophenotype, with a more than two-fold increase in central memory cells in CD8+ 4-1BBζ CAR T-cell products.

**Conclusion:**

Our data indicate that the 4-1BBζ CAR enhances CD8+ TCR-mediated function. This could be beneficial if the TCR targets epitopes on malignant tissues or infectious agents, but detrimental if the TCR targets autoantigens.

**Supplementary Information:**

The online version contains supplementary material available at 10.1186/s12935-023-03171-7.

## Background

Chimeric antigen receptor (CAR) T-cells are genetically engineered T-cells that express the single-chain variable fragment (scFv) of an antibody connected to T-cell intracellular signalling domains. This cellular immunotherapy has dramatically transformed the therapy of CD19+ and BCMA+ haematological malignancies [1–3]. One major advance in CAR design that enhanced in vivo efficacy of CAR T-cells was the evolution of ‘second generation’ CAR endodomains that contained either the CD28 or 4-1BB costimulatory domains in addition to CD3ζ [4, 5]. These costimulatory molecules have significant biological roles, and while they exhibit functional similarities, there are also notable signalling differences between the two second-generation CARs upon scFv engagement with target antigen [6, 7]. These differences can be identified even in the absence of scFv engagement due to the phenomenon of tonic antigen-independent signalling [8]. While antigen-independent tonic signalling was previously thought to be an undesirable process that only influenced some CAR T-cells [8], there is now growing evidence that it occurs universally in CAR T-cells, albeit in varying degrees [9]. Therefore, the biology of these T-cells is fundamentally altered by the integration of a CAR construct.

However, the biology of CAR T-cells as a newly engineered cell ‘subset’ has yet to be comprehensively elucidated. It is unclear whether T-cell receptor (TCR) signalling and functioning is affected by the presence of a chimeric antigen receptor (CAR). CAR T-cells can theoretically mount an immune response to malignant neo-epitopes via their endogenous TCR if the TCR targets the appropriate antigens. While CAR T-cell clonal kinetics have been monitored using TCR sequencing [10], it is unknown whether a CAR T-cell’s endogenous TCR participates in the process of diversifying the anti-malignant immune repertoire during epitope-spreading. In addition, patients treated with anti-CD19 CAR T-cells are at substantial risk of infection due to B-cell depletion, multiple prior chemotherapeutic regimens, pre-CAR T-cell lymphodepletive conditioning, and the immunosuppressive management of immune-related toxicities [11]. Currently, infection is a major cause of non-relapse mortality associated with CAR T-cell therapy making it critical to determine whether CAR T-cells provide the same degree of immunity as unmodified T-cells [11]. Furthermore, any augmented T-cell function may lead to safety concerns in the uncommon case when autoreactive T-cells are administered in patients with pre-existing T-cell mediated autoimmune diseases, or who are given CAR T-cells in conjunction with immune checkpoint blockade. Finally, the risk of graft versus host disease (GVHD) post-allogeneic haematopoietic stem cell transplant may be increased if the CAR T-cells are donor- rather than recipient-derived.

Information regarding TCR-related function in CAR T-cells is limited and our current understanding is based largely on models of GVHD by Ghosh et al. [12]. While this group was concerned primarily with GVHD as a disease complication for patients undergoing allogeneic haematopoietic transplantation, GVHD is also a model of TCR-driven immune responses because major histocompatibility complex (MHC) mismatch is a profound TCR stimulant. In mice treated with allogeneic transplants (B6 into BALB/c) and anti-CD19 donor-derived CAR T-cells, it was identified that there were substantial differences in GVHD which were dependent on the CAR endodomain. In these models, CARs with a 4-1BBζ endodomain caused severe GVHD, allogeneic T-cells transduced with a first-generation CAR (CD3ζ endodomain only) had a minor decrease in GVHD severity, and in contrast, the CD28ζ CAR was associated with a significantly lower rate of GVHD when compared to the 4-1BBζ CAR and control vector [12]. Unfortunately, the only other paper to address TCR-mediated function in the form of GVHD obtained differing results that may be due to an alternative design of CAR construct with mutated immunoreceptor tyrosine-based activation motifs (ITAMS) (CD3ζ X2X) and a different (minor- rather than major-mismatched) model of allogeneic transplantation [13]. This second study compared allogeneic CARs with syngeneic CARs but did not include allogeneic mock-transduced cells to address whether the CAR construct altered T-cell function.

Therefore, this study aimed to assess T-cell function dependent on the endogenous TCR of CAR T-cells by evaluating several aspects relevant to anti-tumour immunity. The SS1 scFv targeting mesothelin (MSLN) was evaluated because it is a clinically relevant scFv, which has been investigated in Phase 1 trials against solid organ malignancies [14, 15]. In addition, MSLN is not detected in the peripheral blood and bone marrow from healthy individuals [16] and therefore has negligible risk of activation via the scFv during expansion or in vitro assays*.* Two common costimulatory domains used in clinical trials, CD28 and 4-1BB, were evaluated in these assays. They were compared to untransduced T-cells as a representation of unmodified T-cells, and ζ-mutant (Mutζ) CAR T-cells, which contain a mutated CD3ζ endodomain with non-functional ITAMS, as a control for the transduction process. T-cell activation was evaluated via the activation marker CD69, cytokine production, degranulation, and proliferation. We evaluated zeta-chain-associated protein kinase 70 (ZAP70), the factor directly downstream of CD3ζ that is responsible for propagating CAR T-cell signalling and likely to be a key determinant in signalling strength [17]. Our results showed increased functionality in CD8+ 4-1BBζ CARs and an altered immunophenotype which has implications for the intended clinical use of these T-cells.

## Methods

### Cell line tissue culture

As MSLN is expressed on pancreatic cancer, MSLN-expressing pancreatic cell lines (AsPC1 and Capan2), as well as a non-MSLN-expressing pancreatic cell line (MIA PaCa2) were chosen for confirming CAR T-cell function. AsPC1, Capan2, MIA PaCa2 cells were obtained from the American Type Culture Collection (ATCC). HEK293T cells, used for retroviral manufacturing, were authenticated by short tandem repeat analysis (CellBank Australia). AsPC1, Capan2 and MIA PaCa2 cell lines were cultured in RPMI 1620 (Gibco), and HEK293T cells in DMEM (Gibco); all culture media was supplemented by 10% (v/v) foetal bovine serum (Bovogen, Australia), 1% (v/v) penicillin–streptomycin (Gibco) and 1% (v/v) GlutaMax (Gibco).

### Generating the AsPC1 MSLN knockout

Complementary oligos constituting a single guide RNA (sgRNA) targeting *MSLN* coding exon 2 with the sequence (5′-3′) CCTGGCTGGAGAGACAGGGC was ligated into the pLKO.1-puro U6 sgRNA BfuA1 2a-H2B-mCherry vector (modified with H2B-2A-mCherry from Addgene plasmid #52628). A single guide RNA targeting the *AAVS1* locus with the sequence (5′-3′) ACCCCACATGGGGCCACTA was used as a control guide. The MSLN or control sgRNA plasmid was co-transfected along with pLV-UbC-eGFP2aCas9 (Addgene #53190) into AsPC1 cells. Cells co-expressing eGFP and mCherry were enriched by fluorescence-activated cell sorting (FACS) and limiting cell dilution performed to derive single cell clones. *MSLN* knockout clones were validated by western blot and flow cytometry.

### CAR plasmid preparation

The SFG retroviral vector backbone was a kind gift from Michel Sadelain (Memorial Sloan Kettering Cancer Centre, New York, USA). Geneblocks for the SS1 CAR constructs with varying endodomains were synthesized (IDT, Iowa, USA) and ligated into the vector backbone. Plasmid preparation of suitable recombinant plasmids was performed by NucleoSpin Plasmid kits (Machery-Nagel) and confirmed by Sanger Sequencing (Australian Genome Research Facility).

### CAR viral preparation

A ‘ping-pong’ amplification strategy was used to generate high-titre CAR-containing retrovirus. The SS1 CAR-containing SFG plasmids were transfected into HEK293T cells along with packaging plasmids: pJK3, pCMVTat and pL-VSV-G. The VSV-G pseudotyped viral supernatants were collected at 48 h, 0.45 μM-filtered and snap-frozen. Subsequently, gibbon ape leukaemia virus producer cells (293 Vec-GALV™, Biovec Pharma) for Moloney murine leukaemia virus-based vectors were transduced with the VSV-G pseudotyped virus. High CAR-expressing producer cells were isolated by FACS after labelling with biotinylated Protein L (#M00097, Genscript) and Streptavidin–phycoerythrin (PE) (#349023, Becton Dickinson, BD). To generate high concentration viral supernatant, SS1 CAR-containing GALV producer cells were plated in 15 cm plates, media was changed at 72 h and viral supernatant collected 24 h later. Viral supernatant was 0.45 μM filtered and snap-frozen.

### CAR T-cell production

CAR T-cell production was adapted from a protocol kindly provided by Xiuyan Wang (Memorial Sloan Kettering Cancer Centre). Blood was collected from three healthy donors under the ethics protocol X18-0050, Sydney Local Health District Ethics (Eastern Zone) Committee. Peripheral blood mononuclear cells (PBMCs) were isolated by collection in sodium heparin cell preparation tubes (#362780, BD) according to manufacturer’s instructions. Any contaminating red cells were lysed with ammonium-chloride-potassium (#A10492, Gibco). PBMCs were either cryopreserved or manufactured immediately. PBMCs were cultured in media consisting of RPMI 1640, supplemented by 10% (v/v) foetal bovine serum, 1% (v/v) penicillin–streptomycin and 1% (v/v) GlutaMax as described above. To remove myeloid subsets, PBMCs were allowed to adhere to a tissue culture plate for a minimum of 3 h, and non-adherent cells harvested from the supernatant. Cells were expanded using CD3/CD28 Dynabeads (#11141D, Thermofisher) at a 3:1 ratio and 100 IU/mL premium grade IL-2 Improved Sequence (#130-097-746, Miltenyi). After 48 h of expansion, cells were ‘spin-oculated’ with SS1 CAR-containing retrovirus in a total volume of 2 mL of media at multiplicities of infection ranging from 4.2–8.9. ‘Spin-oculation’ was at 524 G for 90 min at 32 °C on Retronectin-(#T100B, Takara) coated plates. Cells were expanded in G-Rex plates (Wilson Wolf, USA) for 13 days then cryopreserved before functional analysis.

### Flow cytometry

All phenotyping and functional testing was performed on cells that were rested for 24 h post-thawing to enhance functional responses [18]. Viability was assessed by staining with either Zombie Ultraviolet, Violet or Green (#423107, 423114 or 423111, Biolegend) at a 1:1000 dilution in PBS for 15 min at room temperature. Transduction efficiency and CAR gating was assessed using 0.05 µg/mL biotinylated Protein L (#M00097, Genscript) for 60 min on ice, followed by two washes and then incubation with 0.3 µg/mL streptavidin-APC (#554067, BD Pharmingen). Antibodies to CD4, CD8, CD45RO, CCR7, PD1 and Tim3 (Table 1) were added, and cells stained for 60 min on ice. After washing, the cells were fixed with Cytofix Fixation Buffer (#554655, BD) for 15 min at room temperature, washed again, and events were acquired on the BD LSR Fortessa or BD LSR II flow cytometer. Compensation and analysis were performed using Flowjo version 10.8.1 (Treestar, USA). In multiparameter phenotyping, ‘fluorescence-minus-one’ was used to establish gates for CD45RO, CCR7, PD1 and Tim3 respectively. In staining of Capan2 or AsPC1 MSLN KO, cells were either stained with anti-MSLN antibody, Rat IgG2a isotype control (Table 1) or unstained and fixed as described above.

**Table 1:** Summary of all antibodies used in the study

Target	Use	Clone	Fluorochrome	Working concentration (µg/mL)	Supplier	Catalogue
CD3	IF	UCT1	BB515	1	BD Horizon	564465
CD4	FC	RPA-T4	BV510	1	Biolegend	300546
CD8	FC	RPA-T8	BUV395	0.5	BD Horizon	563795
CD45RO	FC	UCHL1	BB515	2	BD Horizon	564529
CCR7	FC	G043h7	PE-Cy7	0.4	Biolegend	353226
PD1	FC	EbioJ105	PerCP-EF710	0.5	Invitrogen eBioscience	46-2799-42
Tim3	FC	7D3	PE	0.4	BD Pharmingen	563422
ZAP70	FC	1E7.2	PE	1	BD	344635
Mouse IgG1 Kappa Isotype Control	FC	MOPC-21	PE	As appropriate	BD Pharmingen	554680
Phospho ZAP70 (Tyr 493)	FC	A16043E	PE	0.05	Biolegend	396004
CD3ζ	FC	6B10.2	APC	2	Invitrogen eBioscience	396004
CD69	FC	REA824	PE	0.25	Miltenyi	130-112-613
Human IgG1 REA Isotype Control	FC	REA293	PE	As appropriate	Miltenyi	130-113-462
CD107a	FC	REA792	PE	0.3	Miltenyi	130-111-621
Mesothelin	WB	MN1	–	1	Rocklands	200301A88
GAPDH	WB	6C5	–	0.4	Abcam	Ab8245
CD28	Stim	L295	–	1	BD	340975
MSLN	FC	FAB32652P	PE	1	R&D	FAB32652P
Rat IgG2a Isotype Control	FC	54447	PE	1	R&D	IC0006P

IF: immunofluorescence; FC: flow cytometry; WB: western blot; Stim: stimulation

### ZAP70 assays

For total ZAP70 analysis, biotinylated protein L incubation was performed as above, with subsequent streptavidin-APC labelling and staining for CD4 and CD8. The cells were then permeabilized with BD Cytofix/Cytoperm (BD, 554722) for 10 min at 37 °C and washed with Permwash Buffer (BD, 554723). Cells were intracellularly stained with anti-ZAP70 antibody or mouse anti-human IgG kappa isotype control (Table 1) for 60 min on ice, followed by washing and acquisition on the flow cytometer. For phosphorylated ZAP70 (pZAP70) assays, a viability stain was performed with Zombie Ultraviolet for 15 min at room temperature and, after washing, the cells were incubated in reduced serum (2% v/v) RPMI for 1 h at 37 °C. Pervanadate (500 μM, 5 μL) was made from sodium orthovanadate (#sc3540, ChemCruz) as described previously [19], and added to 50 μL of cells. Cells were incubated for 5 min at 37 °C and then 100 μL of Cytofix/Cytoperm added for 10 min at 37 °C. The cells were washed with Permwash Buffer. The cells were subsequently stained with anti-pZAP70 or isotype control, along with antibodies to CD3ζ, CD4 and CD8 for 45 min on ice (Table 1). Cells were washed with Permwash Buffer and then acquired on a flow cytometer. The extent of T-cell signalling via pZAP70 was quantitated using the median fluorescence intensity (MFI) from the stimulated peak on flow cytometry histograms.

### Functional assays

To assess proliferation, CAR T-cells were counted and incubated with CellTrace Violet proliferative dye (#C34571, Invitrogen) for 20 min at room temperature shielded from light. Cells were pelleted and resuspended in fresh complete media. Cells (2 × 10^6^) were then plated at a concentration of 1 × 10^6^ cells/mL in a 12-well plate and stimulated with 1 µg/mL staphylococcal enterotoxin B (SEB) (#S4881, Sigma-Aldrich). As SEB alone does not induce proliferation in a pure T-cell population, the cells were supplemented with 1 µg/mL anti-CD28 L295 (Table 1) [20]. After 5 days, the cells were analysed with Zombie Green viability dye, protein L/streptavidin-APC, anti-CD4 and anti-CD8 antibodies as described above. The ‘percent-divided’ and division index for CAR+ CD4+ and CD8+ T-cells were calculated using the ‘Proliferation Modelling’ function in Flowjo.

For the IFNγ, CD107a and CD69 assays CAR T-cells were plated at a concentration of 1 × 10^6^ cells/mL in a 24-well plate and stimulated with 1 µg/mL SEB or 10 ng/mL of phorbol-12-myristate-13-acetate (PMA) (#P1585, Sigma Aldrich) with 0.4 µg/mL of ionomycin (#I9667, Sigma). For the IFNγ assay, the cells were stimulated for 24 h, and 1 μL/mL monensin (#55472, BD Bioscience) and 1 μL/mL brefeldin (#555029, BD Bioscience) were added for the final 4 h of incubation. For the CD107a assay, the cells were stimulated for 4 h in the presence of monensin. In the CD69 assay, the cells were stimulated for 24 h, then washed, stained with viability dye, protein L/streptavidin-APC, and antibodies to CD4, CD8, CD69 or CD107a (Table 1) followed by fixation as described above. For the IFNγ assay, the cells were fixed and permeabilized with Cytofix/Cytoperm (BD) for 15 min at room temperature followed by washing with Permwash Buffer (BD). The cells were stained with antibodies to IFNγ or the isotype control (Table 1) for a further 60 min on ice before being washed with Permwash Buffer and acquired on a flow cytometer. An isotype control was used to establish gates for CD69, CD107a, IFNγ, ZAP70 and pZAP70-positive cell subsets. Functional assays were performed on each independent donor on different days.

### CAR scFv imaging

CAR T-cells were incubated with 0.05 µg/mL biotinylated Protein L for 45 min on ice, then washed twice with PBS. Cells were fixed with 4% (w/v) Paraformaldehyde Cytofix Fixation Buffer, for 15 min at room temperature and then washed. Cells were stained with 0.3 µg/mL Streptavidin-APC, 4 µg/mL anti-CD3 UCHT1 Brilliant Blue 515 (Table 1) and 0.2 μg/mL 4′,6-Diamidino-2-phenylindole dihydrochloride (DAPI, #D1306, Invitrogen). After washing, cells were resuspended in a 50 µL of Vectashield antifade mounting media (#H-1000-10, Vector Laboratories) and added to a well in an 8-well Ibitreat µ-Slide (#80826, Ibidi). Cells were imaged on a DeltaVision Personal (Applied Precision) microscope using the 40 × objective.

### xCELLigence cytotoxicity assay

Complete RPMI 1640 media was added to the wells of a 96-well PET E-Plate (#300600910, Agilent) and the plate equilibrated in the xCELLigence Real Time Cell Analysis- Single Plate reader (Agilent, California, United States). AsPC1 MSLN knockout (20,000) or Capan2 (10,000) cells were plated in each well. Seeding densities differed between cell lines to generate a satisfactory impedance signal at 24 h. Cells were allowed to adhere for 24 h, then CAR T-cells and untransduced T-cells were added at an effector to target ratio of 2:1, normalised for the differing transduction efficiencies. Target cell impedance was measured over 48 h and the impedance normalised to a value of 1 at the time of co-culture. Real time lysis was calculated relative to the untransduced T-cell control using the following formula:

$$Cytotoxicity = \frac{{100 \times \left( {Average \, Impedance \, Untransduced \, cells - Impedance \, of \, Sample} \right)}}{Average \, Impedance \, Untransduced \, cells}$$

### Western blot

Tumour cell lines were lysed in buffer containing 50 mM Tris–HCL, 150 mM of NaCl, 1 mM Dithiothreitol (DTT), 2 mM Phenylmethylsulfonyl Fluoride, protease inhibitor (1x), 0.5% (v/v) NP-40, 10% (v/v) glycerol, 2 mM MgCl_2_, 0.5% (v/v) Triton X-100 and H_2_O. DTT was diluted 1:10 in SDS NuPAGE loading buffer (#NP0007, Invitrogen) and 10 μL added to each sample and incubated at 80 °C for 5 min. Samples were added to NuPAGE 4–12% BisTris Gel (#NP0035BOX, Invitrogen) along with Precision Dual Colour Protein Standard (#161-0374, Biorad). The gel was run for 130 V for 90 min. The gel was transferred to a PVDF membrane (#IVPH00010, Immobilon), blocked with 5% (w/v) milk powder blocking buffer in phosphate buffered saline with 0.1% (v/v) Tween (PBST) for 30 min. The membrane was then incubated with the MN1 anti-MSLN antibody (Table 1) overnight at 4 °C. The membrane was washed three times with PBST and incubated with donkey anti-mouse secondary antibody (#AP192P, Chemicon) for 2 h. The membrane was washed 3 times with PBST. The membrane was treated with SuperSignal West Pico PLUS Chemiluminescent Substrate (#34580, Thermo Fisher) and visualised on a Biorad Chemidoc Imaging system. The membrane was stripped with Reblot Plus Strong Solution (#2504, Merck Millipore) and then reassessed using mouse anti-GADPH 6C5 antibody (Table 1) overnight at 4 °C. The membrane was processed as described above with the same anti-mouse secondary antibody and imaged again.

### Statistical analysis

Statistical analysis was performed using GraphPad Prism v.9.4.1 (GraphPad Software, California, USA). Comparisons between each cell product were made by one-way ANOVA. Statistical corrections for multiple comparisons were performed to reduce type 1 error using Tukey’s multiple comparison test.

## Results

### SS1 CAR endodomain composition influences CAR T-cell immunophenotype

**Fig. 1:**
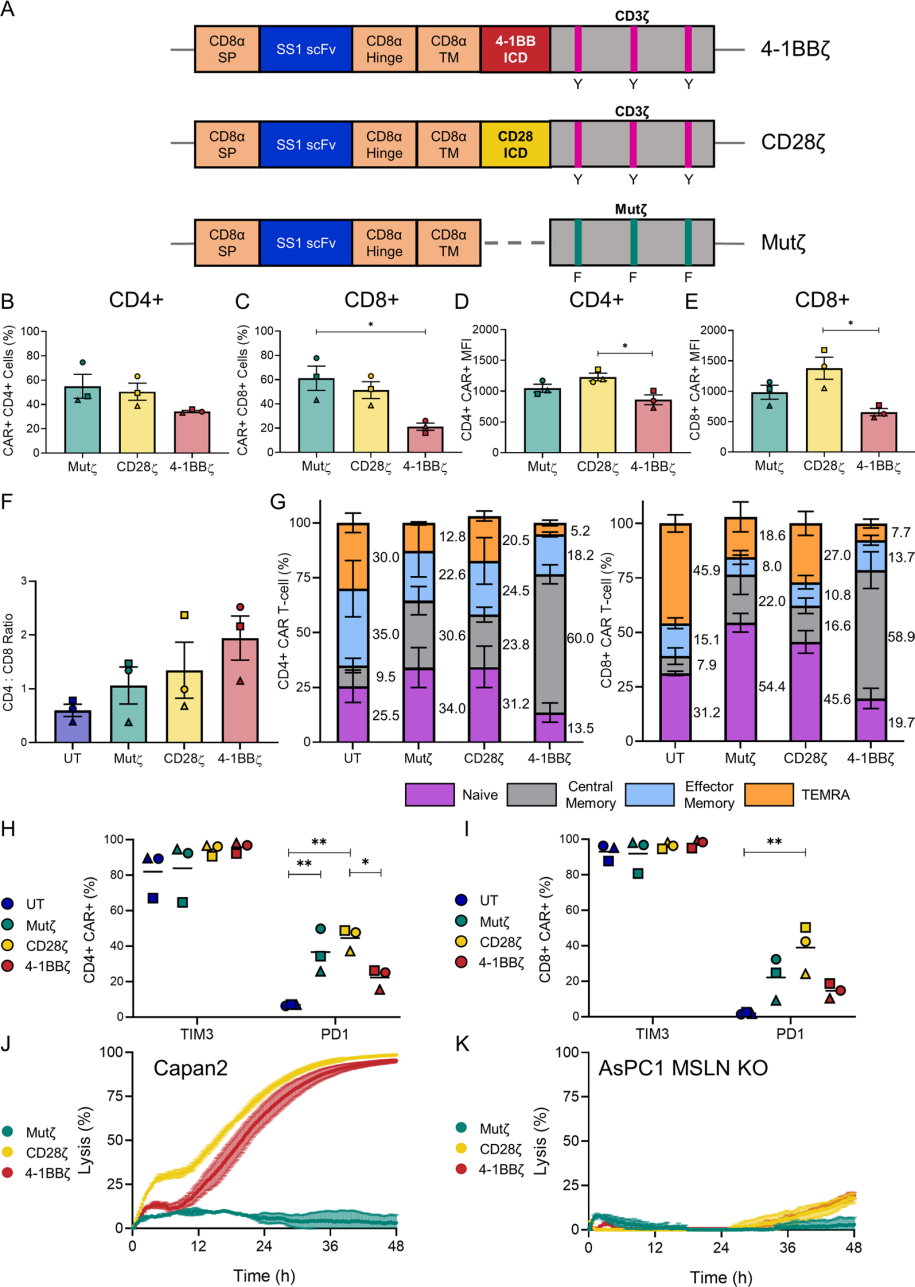
SS1 CAR T-cells with varying endodomains exhibit differing immunophenotypes. **A** Schematic of 4-1BBζ, CD28ζ and Mutζ SS1 CAR constructs expressed via Moloney murine leukaemia retroviral vectors. SP: Signal peptide; scFv: Single chain variable fragment; TM: Transmembrane; ICD: Intracellular domain. Magenta and teal strips denote functional and non-functional immunoreceptor tyrosine-based activation motifs (ITAMs) respectively: Y: tyrosine; F: phenylalanine residues. **B**, **C** Transduction efficiency as measured by Protein L of **B** CD4+, and **C** CD8+ T-cell products. **D**, **E** CAR expression in **D** CD4+, and **E** CD8+ CAR T-cells measured by biotinylated protein L and streptavidin-APC. **F** The CD4: CD8 ratio of each cell product; UT: untransduced. **G** Stacked bar graphs of mean memory cell composition (totaling 100%) in CD4+ and CD8+ cell products phenotyped with anti-CCR7 and anti-CD45RO antibodies. In **B**–**G**, the mean and standard error of the mean (SEM) are indicated. Values are presented as averages of 3 donors and therefore the total values differ subtlety. **H**, **I** Expression of exhaustion markers TIM3 and PD1 in **H** CD4+, and **I** CD8+ CAR T-cell products; the mean is indicated. Experiments were performed with n = 3 independent donors, each analysed on different days, in **B**–**F** and **H**, **I**; each donor is distinguished by a unique symbol. Comparisons were made between all cell products by one-way ANOVA with Tukey’s correction for multiple comparisons; only statistically significant differences are indicated (*p < 0.05, **p < 0.01, ***p < 0.001). **J**, **K** xCELLigence real-time cytotoxicity assay of SS1 cell products against **J** Capan2, and **K** AsPC1 MSLN KO at a normalized effector to target ratio of 2:1. Lysis is normalized to untransduced T-cells and data are representative of 3 independent experiments with independent donors performed on different days. In **J**, **K** the mean and standard deviation are indicated

CAR T-cell products were manufactured using Moloney murine leukaemia virus-based retroviral vectors containing the SS1 scFv, the CD8α signal peptide, hinge and transmembrane regions (Fig. 1A) and different intracellular domains (ICDs). The 4-1BBζ and CD28ζ ICDs were investigated as these are utilized in current Food and Drug Administration-approved CAR T-cell therapies. Tyrosine (Y) residues in the CD3ζ ITAMs were mutated to phenylalanine (F) residues to prevent phosphorylation and signalling as a control (Mutζ, Fig. 1A) [9]. The purpose of the Mutζ cell product was to control the entire process of CAR T-cell manufacturing, viral transduction and in vitro expansion, without generating any constitutive tonic signalling via the CAR construct. The Mutζ control has an advantage over controls that lack an intracellular domain, because it controls for how intracellular structure might affect the distance between adjacent CAR molecules, controls for how intracellular structure might interact and approximate with the endogenous T-cell signalling molecules, and is a similar transgene size compared to the functional CD28ζ and 4-1BBζ CAR constructs.

Functional and phenotypic analyses were performed by flow cytometry using the gating strategy outlined in Additional file 1: Fig. S1A to analyse live, singlet CD4+ and CD8+ T-cells. No reporter gene was included so that any functional changes could be attributed solely to the CAR construct. Therefore, CAR T-cells were identified using two gating strategies. Biotinylated protein L was used to bind to the scFv which is present on CAR T-cells but not on normal T-cells, and was detected using streptavidin-APC (Additional file 1: Fig. S1B). However, this strategy was found to be unsuitable for intracellular signalling assays investigating pZAP70. Instead, when evaluating pZAP70, intracellular CD3ζ antibody staining was used to identify CAR T-cells, which have increased CD3ζ levels compared to untransduced T-cells (Additional file 1: Fig. S1C). All CARs, including the Mutζ CARs were identified by intracellular CD3ζ, albeit with reduced resolution compared to protein L detection due to endogenous CD3ζ expression.

The transduction efficiency as measured by Protein L staining was noted to be lower in 4-1BBζ CARs (Fig. 1B, C), although this was only statistically significant when CD8+ 4-1BBζ CAR T-cells were compared to Mutζ CD8+ T-cells. Expression of the CAR scFv measured by protein L-APC was also significantly lower in 4-1BBζ CARs compared to CD28ζ CAR T-cells (Fig. 1D, E). The transduction efficiency for 4-1BBζ CAR T-cells could not be further improved with additional virus, and is possibly due to the gradual downregulation of gamma retrovirally-expressed 4-1BBζ CARs during the manufacturing process [21]. The CD4:CD8 ratio varied between cell products, with a greater proportion of CD4+ T-cells in the 4-1BBζ CAR T-cells compared to untransduced T-cells (Fig. 1F) similar to previous reports of retrovirally-expressed 4-1BBζ CARs, although this did not reach statistical significance [21]. All subsequent flow analyses were gated on CAR-expressing populations.

Next, the memory phenotype of SS1 CAR T-cells was evaluated with antibodies to CCR7 and CD45RO in the three healthy human donors. 4-1BBζ CAR T-cells had an enrichment in central memory phenotype with a corresponding reduction in naïve, effector memory and effector memory T-cells re-expressing CD45RA (TEMRA) (Fig. 1G, Additional file 1: Fig. S1D). CD4+ 4-1BBζ cell products had a significantly higher mean percentage of central memory cells (63.0 ± 4.3% SEM compared to CD4+ untransduced T-cells (9.5 ± 3.3%), Mutζ (30.6 ± 6.5%) and CD28ζ cell products (23.4 ± 3.4%) (*p* < 0.001, *p* = 0.004, *p* = 0.001 respectively). Similarly, CD8+ 4-1BBζ cell products had a higher mean percentage of central memory cells (58.9 ± 8.5% SEM) compared to CD8+ untransduced T-cells (7.9 ± 3.8%), Mutζ (22.0 ± 9.1%) and CD28ζ cell products (16.6 ± 3.5%) (*p* = 0.003, *p* = 0.02, *p* = 0.009 respectively). The findings are consistent with changes in memory status noted with unstimulated FMC63-derived CD19 CARs with differing endodomains [9].

T-cell immunoglobulin and mucin domain 3 (TIM3) and programmed cell death protein 1 (PD1), are both associated with exhaustion in vivo but they can also be markers of activation in vitro [22–24]*.* TIM3 was found to be highly expressed (> 60%) in all cell products (Fig. 1H, I). 4-1BBζ CARs expressed lower PD1 compared to their CD28ζ counterparts (mean ± SEM: 22.3 ± 3.4% vs. 44.6 ± 3.7% for CD4+ CARs, *p* = 0.03; mean ± SEM 14.7 ± 2.4% vs. 39.0 ± 17.7% for CD8+ CARs, *p* = 0.05 respectively). All virally transduced cells had an increased level of PD1 compared to untransduced T-cells, although this was only statistically significant for CD4+ Mutζ (*p* = 0.005), and CD4+ and CD8+ CD28ζ CAR T-cells (*p* = 0.001,* p* = 0.005, respectively). While these differences may be associated with exhaustion and/or activation levels due to the different constructs, elucidation of the specific functional changes associated with changes in these molecules was beyond the scope of the current study.

Despite immunophenotypic differences, CD28ζ and 4-1BBζ CAR T-cells demonstrated similar efficacy in real-time cytotoxicity assays against the pancreatic cancer cell line, Capan2, which expresses MSLN natively (Fig. 1J) with > 95% tumour cell lysis at 48 h. Both CD28ζ and 4-1BBζ CARs demonstrated only minor off-target lysis against the AsPC1 MSLN knockout cell line (Fig. 1K). The inactive Mutζ CAR control did not demonstrate any lysis against either cell line (Fig. 1J, K). This pattern was noted with all three donors (Additional file 2: Fig. S2A, B). The presence or absence of MSLN in both cell lines was confirmed by western blot (Additional file 2: Fig. S2C).

**Fig. 2:**
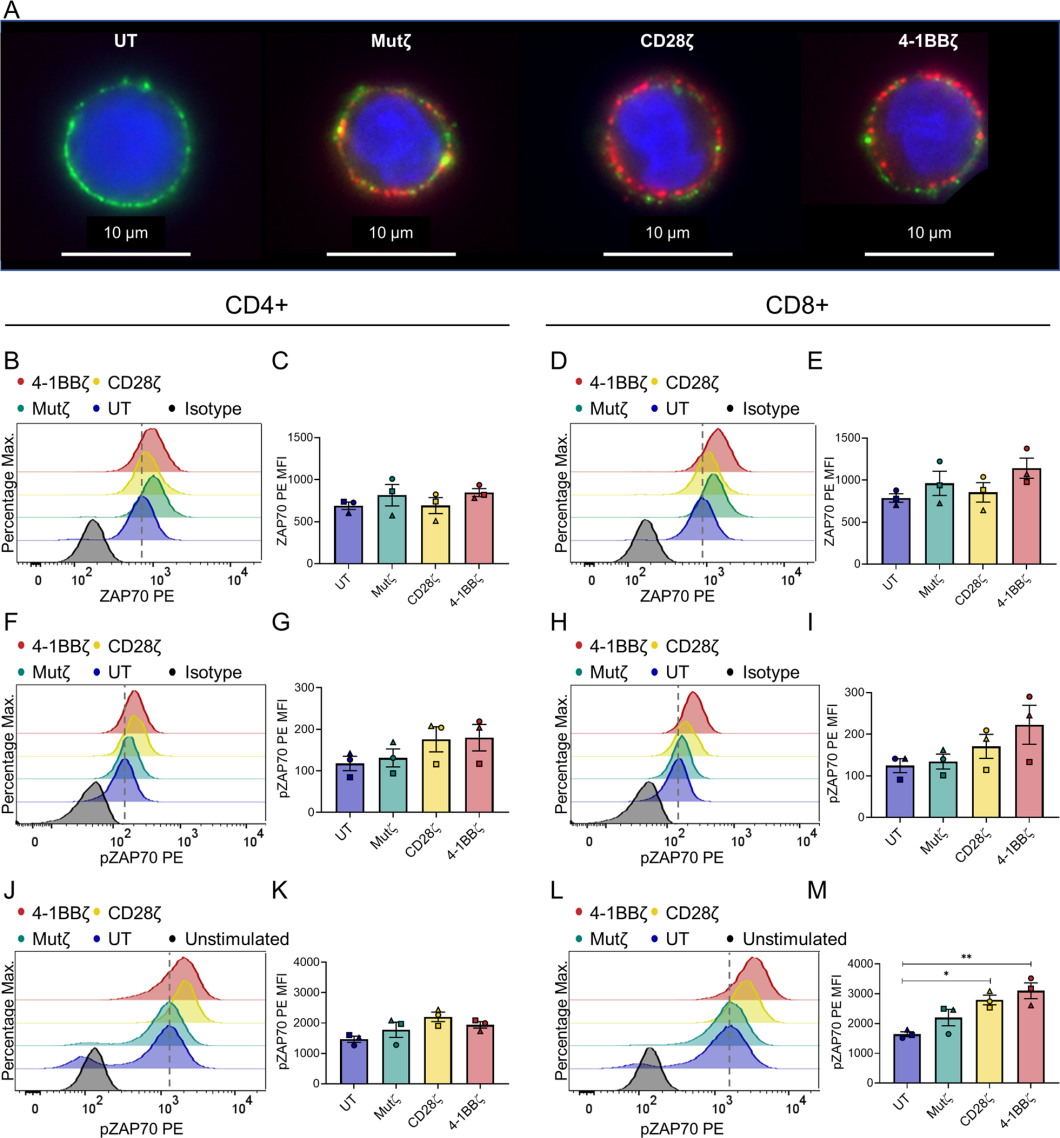
CD8+ SS1 CAR T-cells have increased pZAP70 after pervanadate stimulation. **A** CAR scFv distribution in the cell membrane in untransduced cells (UT), Mutζ, CD28ζ and 4-1BBζ CARs; CD3 (Green), CAR scFv (Red), DAPI (Blue). **B**–**E** Relative levels of ZAP70 expression by flow cytometry. Histograms of ZAP70 in a representative donor’s **B** CD4+, and **D** CD8+ cell products. Bar graphs of ZAP70 expression from three donors measured by MFI in **C** CD4+, and **E** CD8+ cell products. **F**–**M** Relative levels of phosphorylated ZAP70 (pZAP70) from three donors by flow cytometry. Histograms of pZAP70 in a representative donor’s **F** CD4+, and **H** CD8+ cell products at rest and **J**–**L** after stimulation with pervanadate. Bar graphs of pZAP70 activation measured by MFI in **G** CD4+, and **I** CD8+ cell products at rest, and **K**, **M** after stimulation with pervanadate. The modal peak of untransduced T-cells is indicated by the dashed line in the histograms. Bar graphs are of n = 3 independent donors analysed on different days; each donor is distinguished using a unique symbol. Data in bar graphs represent mean ± SEM. Comparisons were made between all cell products by one-way ANOVA with Tukey’s correction for multiple comparisons; only statistically significant differences are indicated (*p < 0.05, **p < 0.01)

These data confirm that CAR endodomain composition is associated with differences in transduction efficiency, CD4:CD8 ratio, memory phenotype and expression of checkpoint molecules, consistent with earlier reports with other scFvs [9, 21]. However, despite these phenotypic differences, both SS1-CD28ζ and 4-1BBζ CARs were highly cytotoxic in vitro, again, consistent with previous reports [25, 26].

### CD8+ SS1 CAR T-cells with functional endodomains have enhanced signalling responses to pervanadate

Next, imaging of CAR scFv distribution was performed to determine whether there is any spontaneous CAR clustering which might predispose to tonic endodomain signalling [8], using biotinylated protein L labelling of scFvs and CD3ε labelling of the membrane. Uniform distribution of the CAR protein at the cell membrane was observed, suggestive of lower levels of tonic signalling in the SS1 CAR, compared to other CAR constructs described in the literature, such as the anti-GD2 CAR (Fig. 2A) [8]. While imaging provided a gross assessment of scFv distribution, flow cytometry of total ZAP70 and pZAP70 levels was performed to provide a quantitative assessment of tonic signalling. ZAP70 was chosen as it is the first signalling molecule downstream of CD3ζ, and, unlike CD3ζ, is not affected by differences in transduction efficiency or CAR expression. There were no differences in ZAP70 protein levels in CD4+ cell products (Fig. 2B, C). There was a trend towards an increase in ZAP70 protein in CD8+ 4-1BBζ CAR T-cells compared to CD8+ untransduced T-cells (mean ± SEM MFI of 1142 ± 121 vs. 787 ± 51) although this did not reach statistical significance (Fig. 2D, E). Furthermore, CD4+ and CD8+ 4-1BBζ CAR T-cells both exhibited a trend of increased basal pZAP70 compared to untransduced T-cells (mean ± SEM MFI of 180 ± 32 vs. 118 ± 17 for CD4+ T-cells; and 223 ± 47 vs. 124 ± 17 for CD8+ T-cells) although neither of these were statistically significant (Fig. 2F–I). These results suggested that SS1 CARs with functional endodomains exhibit only low levels of tonic signalling via ZAP70.

Pervanadate, a protein tyrosine phosphatase inhibitor that enhances tyrosine phosphorylation, was used as a T-cell stimulant to examine signalling [27, 28]. In CD4+ T-cells, the CAR endodomain did not consistently alter the pZAP70 levels after stimulation compared to untransduced or Mutζ cells (Fig. 2J, K). However, in CD8+ T-cells, higher levels of pZAP70 were detected in pervanadate-stimulated 4-1BBζ CARs compared to their untransduced counterparts (mean ± SEM MFI of 3104 ± 266 vs. 1643 ± 81, *p* = 0.005) (Fig. 2L, M). A similar result was noted in CD8+ CD28ζ CARs compared to untransduced T-cells (mean ± SEM MFI of 2793 ± 162 vs. 1643 ± 81, *p* = 0.02). This demonstrated that the presence of a functional CAR endodomain enhanced the signalling response to pervanadate in CD8+ SS1 CAR T-cells.

### CD8+ 4-1BBζ CARs T-cells proliferate more after staphylococcal enterotoxin B stimulation

**Fig. 3:**
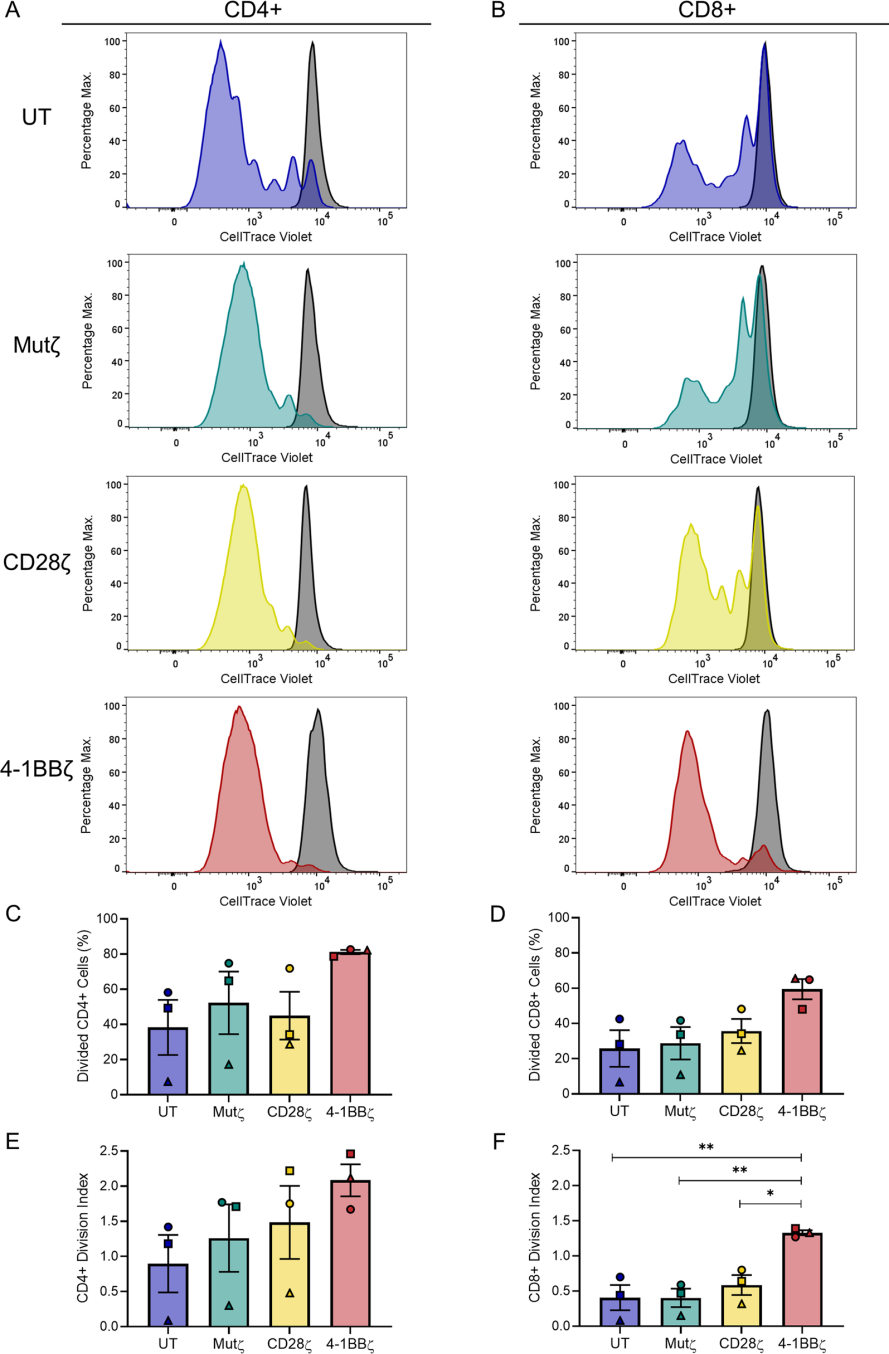
CD8+ 4-1BBζ CAR T-cells exhibit increased proliferation after stimulation with SEB. **A**, **B** Representative flow cytometry histograms of proliferation of **A** CD4+, and **B** CD8+ cell products labelled with CellTrace Violet and stimulated with staphylococcal enterotoxin B for 5 days. The grey histogram indicates the unstimulated control. **C**, **D** Bar graphs of the percentage of divided **C** CD4+, and **D** CD8+ cell products. **E**, **F** Bar graphs of the division index (the average number of divisions) in **E** CD4+, and **F** CD8+ cell products. Experiments were performed on different days for three independent donors; each donor is distinguished using a unique symbol. Data represents mean ± SEM. Comparisons were made between all cell products by one-way ANOVA with Tukey’s correction for multiple comparisons; only statistically significant differences are indicated (*p < 0.05, **p < 0.01)

An increase in TCR signalling measured by pZAP70 is predicted to have downstream functional effects [29]. First, proliferation was assessed with staphylococcal enterotoxin B (SEB), which acts on the TCR variable β (Vβ) domain present in some T-cell Vβ families. An anti-CD28 antibody was added to provide additional costimulation in the absence of antigen presenting cells [20]. As the Vβ repertoire differs between individuals, there was substantial inter-donor variability and a relative depletion of CD8+ T-cells was noted in 2 donors, which may be attributed to activation-induced cell death. Unlike previously reported studies [30], the SS1 CARs did not exhibit any basal proliferation without stimulation (Fig. 3A, B). The percentage of divided cells was increased in 4-1BBζ cell products compared to untransduced T-cells and Mutζ T-cells although this did not reach statistical significance (Fig. 3A–D). Among the CD4+ T-cell products, a mean ± SEM of 81.2 ± 1.2% of 4-1BBζ CAR T-cells had undergone cell division compared to 38.3 ± 15.7% of CD4+ untransduced T-cells and 52.3 ± 17.8% of Mutζ CAR T-cells (Fig. 3C). Among the CD8+ T-cell products, a mean ± SEM of 59.5 ± 5.7% of 4-1BBζ CAR T-cells had undergone division compared to 25.8 ± 10.4% of CD8+ untransduced T-cells and 28.8 ± 9.2% of Mutζ CAR T-cells (Fig. 3D). The division index, which is the average number of divisions per cell, was higher 4-1BBζ CARs, and this difference was statistically significant when considering CD8+ T-cell products (Fig. 3E, F). The mean ± SEM division index for CD8+ 4-1BBζ cells was 1.3 ± 0.0 compared to 0.4 ± 0.2 for untransduced CD8+ T-cells, 0.4 ± 0.1 for Mutζ T-cells and 0.6 ± 0.1 for CD28ζ CAR T-cells (*p* = 0.005, *p* = 0.005, and *p* = 0.02 respectively).

### CD8+ 4-1BBζ CARs have increased IFNγ production after staphylococcal enterotoxin B stimulation

**Fig. 4:**
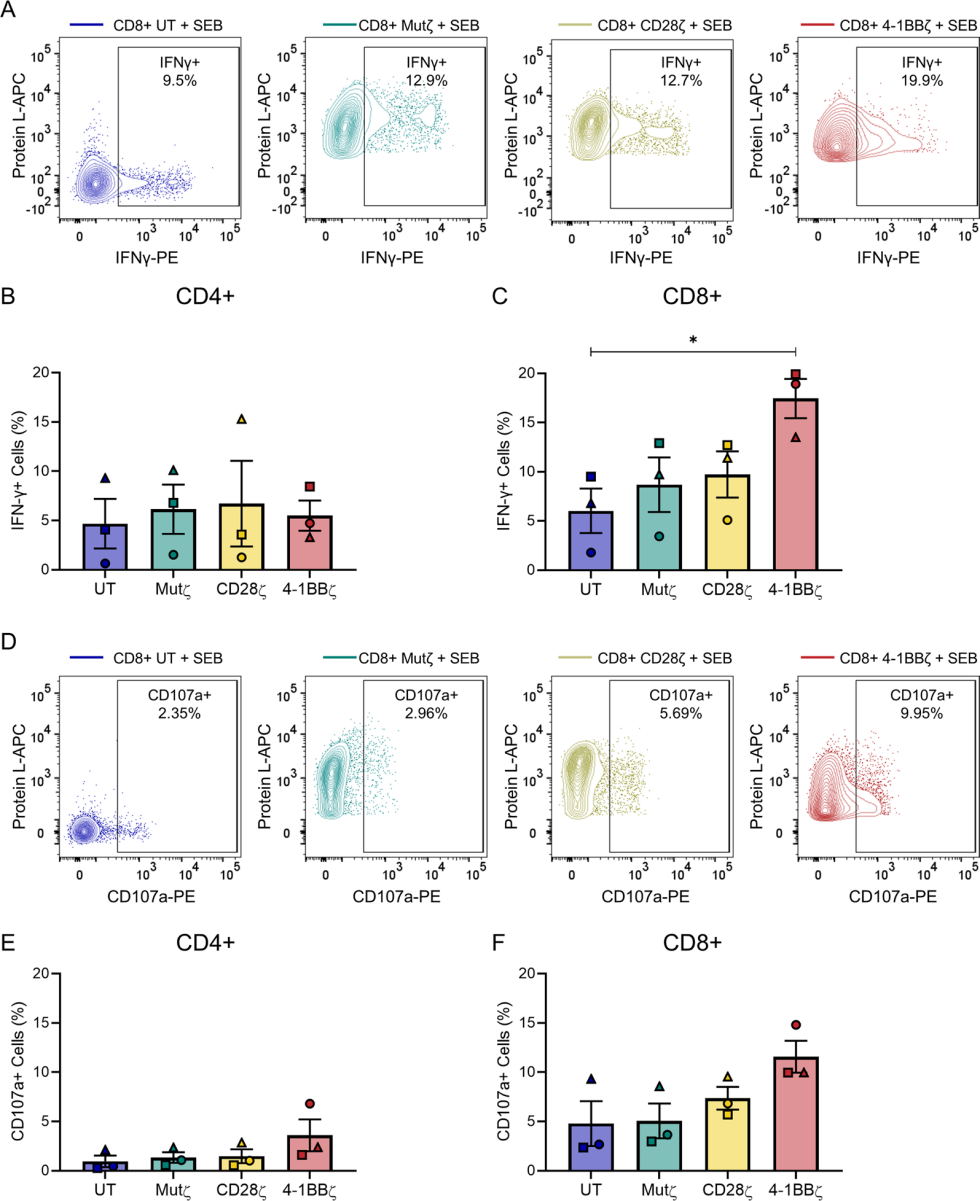
SEB-stimulated CD8+ 4-1BBζ CAR T-cells produce more IFNγ compared to untransduced T-cells. **A** Intracellular IFNγ production 24 h after SEB stimulation in untransduced, Mutζ, CD28ζ and 4-1BBζ CD8+ CAR T-cell products in a representative donor. **B**, **C** Box plot of intracellular IFNγ-production in **B** CD4+, and **C** CD8+ CAR T-cell products. **D** Degranulation measured by surface CD107a expression 4 h after SEB stimulation in untransduced, Mutζ, CD28ζ and 4-1BB CD8+ CAR T-cell products in a representative donor. **E**, **F** Box plot of CD107a expression in **E** CD4+, and **F** CD8+ cell products. Experiments were performed with n = 3 independent donors, each on different days; each donor is distinguished using a unique symbol. The mean and SEM are indicated. Comparisons were made between all cell products by one-way ANOVA with Tukey’s correction for multiple comparisons; only statistically significant differences are indicated (*p < 0.05)

The production of IFNγ as a key cytotoxic cytokine was also assessed in CAR T-cells stimulated by SEB for 24 h (Fig. 4A–C). There was no difference in IFNγ production in stimulated CD4+ T-cell products (Fig. 4B). However, the mean percentage of IFNγ-producing cells was higher in CD8+ 4-1BBζ CARs (mean ± SEM of 17.4 ± 2.0%) compared to CD8+ untransduced T-cells (6.0 ± 2.3%, *p* = 0.04), Mutζ CAR T-cells (8.7 ± 2.8%, *p* = 0.11), and CD28ζ CAR T-cells (9.7 ± 2.4%, *p* = 0.18) (Fig. 4A, C). Degranulation was then assessed by the surface levels of lysosomal-associated membrane protein (CD107a) (Fig. 4D–F). There was no substantial difference in CD107a levels in CD4+ T-cells (Fig. 4E). The percentage of surface CD107a detected in CD8+ 4-1BBζ CAR T-cells was higher compared to CD8+ untransduced T-cells (mean ± SEM 11.6 ± 1.6 vs. 4.8 ± 2.3%) (Fig. 4D, F) but this did not reach statistical significance (*p* = 0.09). While the same difference was not statistically significant with CD107a expression, this could be explained by the lower sensitivity in detecting degranulation in our assay compared to either IFNγ expression or CD69 expression, making it more difficult to appreciate the differences between vectors.

### CD8+ 4-1BBζ CARs have increased CD69 expression after staphylococcal enterotoxin B stimulation

**Fig. 5:**
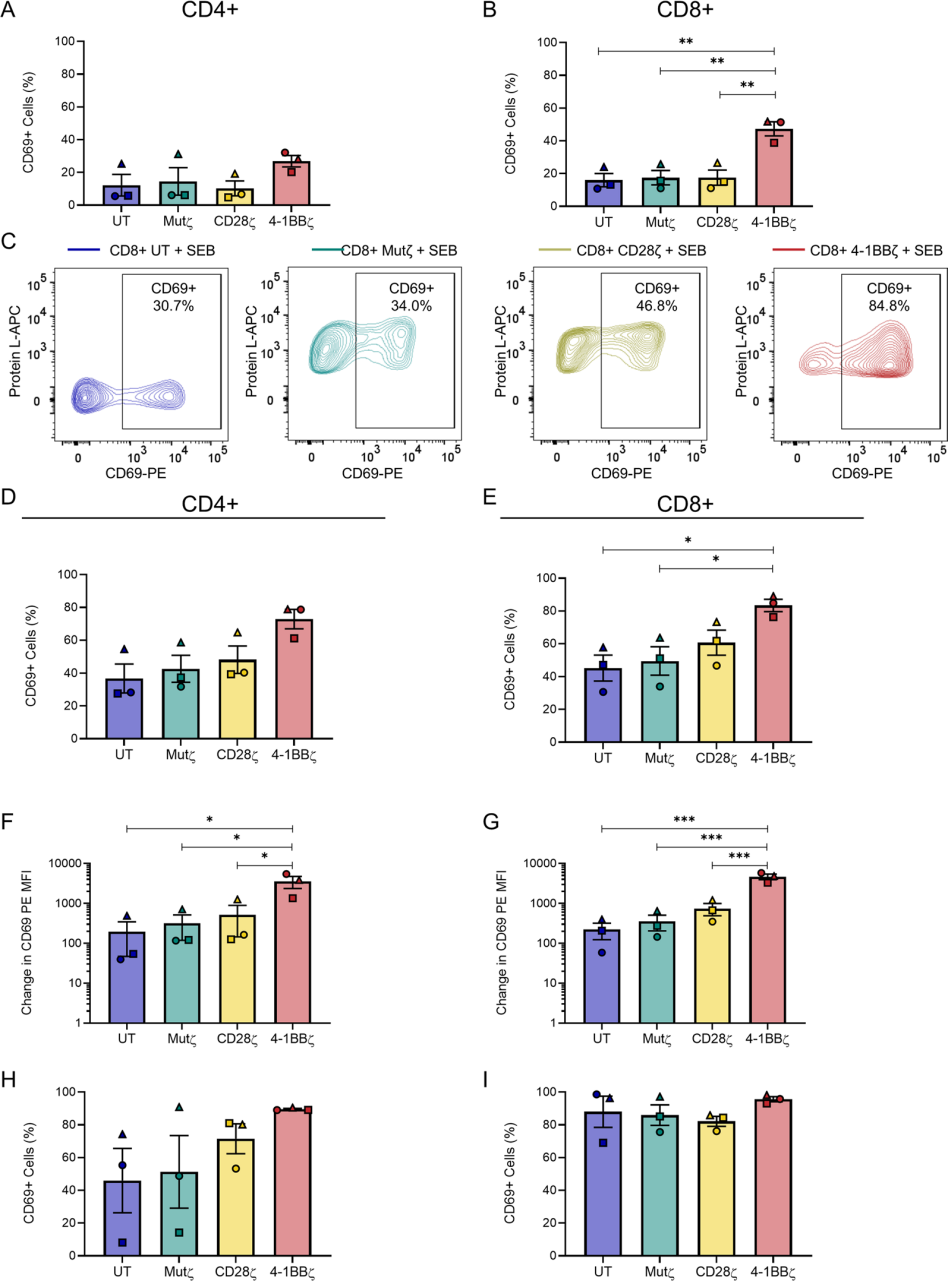
CD69 activation is increased in CD8+ 4-1BBζ CAR T-cells. **A**, **B** Bar graphs of the percentage of CD69 + cells in resting **A** CD4+, and **B** CD8+ cell products. **C** Flow plots of CD69 expression 24 h after SEB stimulation in untransduced, Mutζ, CD28ζ and 4-1BBζ CD8+ CAR T-cell products in a representative donor. **D**, **E** Bar graphs of the percentage of CD69+ cells in **D** CD4+, and **E** CD8+ CAR T-cell products after 24-h stimulation with SEB. **F**, **G** The change in CD69 expression measured by the change in MFI-PE in **F** CD4+, and **G** CD8+ cell products after 24-h stimulation with SEB. (H-I) Bar graphs of the percentage of CD69-positive in **H** CD4+, and **I** CD8+ CAR T-cell products after 24-h stimulation with PMA/Ionomycin. Experiments were performed with n = 3 independent donors, each on different days; each donor is distinguished using a unique symbol. The mean and SEM are indicated. Comparisons were made between all cell products by one-way ANOVA with Tukey’s correction for multiple comparisons; only statistically significant differences are indicated (*p < 0.05, **p < 0.01, ***p < 0.001)

CD69 is a transmembrane C-type lectin and was examined as an early marker of T-cell activation. No significant difference in the percentage of CD69+ CD4+ cells was noted at rest (Fig. 5A). However, there was a significant difference in the percentage of CD69 + CD8+ T-cells at rest (Fig. 5B, C): 47.3 ± 4.3% of CD8+ 4-1BBζ CAR T-cells were CD69 + compared to 16.0 ± 4.1% of CD8+ untransduced T-cells (*p* = 0.004), 17.5 ± 4.4% of CD8+ Mutζ CAR T-cells (*p* = 0.005) and 17.5 ± 4.6% of CD8+ CD28ζ CAR T-cells (*p* = 0.005). After 24-h stimulation with SEB, 72.9 ± 5.9% of CD4+ 4-1BBζ CAR T-cells were CD69 + compared to 36.8 ± 8.9% of CD4+ untransduced T-cells (*p* = 0.05), 42.6 ± 8.2% of CD4+ Mutζ CAR T-cells (*p* = 0.10) and 48.2 ± 8.3% of CD4+ CD28ζ CAR T-cells (*p* = 0.20) (Fig. 5D). Statistically significant differences were noted among CD8+ T-cell products after SEB stimulation; 83.4 ± 3.7% of CD8+ 4-1BBζ CAR T-cells were CD69 + compared to 45.2 ± 7.9% of CD8+ untransduced T-cells (*p* = 0.02), 49.5 ± 8.6% of CD8+ Mutζ CAR T-cells (*p* = 0.04), and 60.6 ± 7.7% of CD8+ CD28ζ CAR T-cells (*p* = 0.19) (Fig. 5E). However, when the change in CD69 level was assessed before and after stimulation by measuring the increase in CD69 MFI, there was a statistically significant difference for both CD4+ and CD8+ 4-1BBζ CAR T-cells compared to all other cell products (Fig. 5F, G).

To determine if this was a global effect seen with all T-cell stimulants, CAR T-cells were also stimulated with phorbol myristate acetate (PMA) and ionomycin for 24 h. These stimulants act further downstream of TCR signal transduction on protein kinase C and calcium flux respectively. Increased variability between donors with these stimulants and a robust response in all CD8+ cell products was noted with PMA/Ionomycin. In contrast to SEB, no statistically significant differences in either the percentage of CD69+ cells (Fig. 5H, I) or in the change in CD69 expression by MFI PE (Additional file 3: Fig. S3A, B) were detected. The enhanced activation of CD8+ 4-1BBζ CAR T-cells appeared to be SEB-specific.

## Discussion

CAR T-cells are now routine therapies for CD19+ and BCMA+ haematological malignancies. While T-cell function and signalling has been extensively studied in CAR T-cells stimulated via their scFv, there is no comprehensive assessment of T-cell function in CAR T-cells that have been stimulated via their TCR, warranting further investigation. For example, Kawalekar et al. identified that when stimulated via their scFv, 4-1BBζ CAR T-cells had increased persistence, central memory phenotype, oxidative metabolism and mitochondria biogenesis compared to CD28ζ CAR T-cells [31]. In the experiments of Kawalekar et al., the CAR T-cells were stimulated with magnetic beads bound with anti-CD19 idiotype or MSLN-Fc. CAR T-cell stimulation via their TCR was not performed. Additional studies have demonstrated that tonic signalling 4-1BBζ anti-GD2 CAR T-cells tested in in vivo leukaemic models have reduced exhaustion, better persistence and superior anti-tumour outcomes compared to their CD28ζ CAR T-cell counterparts. However, our study is the first to our knowledge that reveals CD8+ 4-1BBζ CAR T-cells consistently demonstrate enhanced proliferation, increased activation markers and cytokine production compared to untransduced T-cells when stimulated via the TCR with SEB. These findings cohere with the literature relating to ex vivo expansion of anti-CD19 and anti-CD22 4-1BBζ CAR T-cells [5, 32].

The enhanced functional responses to SEB were more prominent in CD8+ 4-1BBζ CAR T-cells compared to CD4+ 4-1BBζ CAR T-cells. There are two probable contributory explanations. First, 4-1BB has a more prominent biological role in CD8+ T-cells compared to CD4+ T-cells [33–37]. The second explanation is that functional differences in CD8+ 4-1BBζ CAR T-cells could also be related to our CAR construct design which utilises the CD8α hinge and transmembrane domain and dimerisation with endogenous CD8α/β could lead to a higher propensity for stronger signalling in CD8+ CAR T-cells [38, 39].

Our data suggest two possible mechanisms by which 4-1BBζ endodomains enhance TCR-mediated function: increased signalling or phenotypic changes such as the demonstrated enrichment of the central memory T-cell phenotype. First, the differing immunophenotype of CD8+ 4-1BBζ CAR T-cells could be responsible for the enhanced performance in functional assays. For example, the central memory T-cell population was substantially greater in 4-1BBζ CAR T-cells which could lead to a lower risk of anergy and perhaps better functional responses to stimulation. Another immunophenotypic difference that has been previously described in 4-1BBζ CARs is the heightened degree of MHC Class II expression [9], which could potentially lead to an amplified response to SEB.

The second possibility is that different signalling dynamics are contributing to enhanced function. Imaging and resting analyses of pZAP70 did not indicate heightened levels of tonic ZAP70-mediated signalling. This differs with prior observations of tonic signalling in SS1 CAR T-cells, although differences could be due to the alternative transmembrane domain and hinge regions used in our construct [30], as these regions play critical roles in generating immune synapses [40]. Of interest, higher levels of pZAP70 signalling after pervanadate stimulation were noted in CD8+ SS1 CARs with functional endodomains compared to untransduced T-cells. Elevated levels of pZAP70 have been associated elsewhere with stronger and more stable TCR ligation from either high affinity or higher levels of antigen [29], which in turn is associated with greater downstream T-cell signalling [41], proliferation [42], CD69 expression [41], granzyme B and IFNγ production [43], similar to what we identified. However, the elevated levels of pZAP70 in pervanadate-stimulated CD8+ CD28ζ CAR T-cells was not associated with increased functional responses from CD8+ CD28ζ CARs in the other assays, making this unlikely to be the sole explanation for differences in function. Notably, 4-1BB and downstream tumour necrosis factor receptor associated factor (TRAF) signalling is not incorporated in our analyses, and it is possible that tonic signalling is occurring via these pathways, as described elsewhere [21]. Indeed, TRAF-dependent signalling may explain the increased resting 4-1BBζ CAR CD69 expression and the central memory enrichment in the absence of any interaction with target antigen or tonic ZAP70-mediated signalling [21], and may also influence the 4-1BBζ CAR’s response to SEB. Notably, neither of these two proposed mechanisms: differing signalling dynamics or the differing immunophenotype, are mutually exclusive.

Unexpectedly, there was no difference among cell products in CD69 levels following PMA/ionomycin stimulation. However, the differences between the results with SEB and PMA/ionomycin may provide mechanistic insights. The early activation marker, CD69, is induced by the transcription factor activated-protein-1 (AP-1) [44]. Of note, the increased CD69 from 4-1BB signalling is likely via tumour necrosis factor receptor associated factors (TRAFs), leading the increased expression of AP-1 as well as another key transcription factor, nuclear factor kappa-light-chain-enhancer of activated B-cells (NF-ΚB) [45]. In an analogous manner, PMA, via protein kinase C, leads to increased expression of both NF-ΚB and AP-1 [46]. However, PMA is a potent stimulant of protein kinase C, and may lead to maximal activation of these transcriptional factors, such that no additive effect of 4-1BB signalling may be detected. Indeed, experiments using NF-ΚB and AP-1 reporter cell lines show that PMA/ionomycin lead to substantially greater expression of both NF-ΚB and AP-1 compared to either 4-1BB signalling or plate-bound anti-CD3 antibodies used as control [47]. While the reporter cell experiments did not compare PMA/ionomycin with SEB, in T-cell functional assays that do compare PMA/ionomycin with SEB, it is common for CD69 [48], as well as intracellular cytokines [49–51], to be higher with PMA/ionomycin compared to SEB. Therefore, it is conceivable that SEB, especially in the absence of any antigen-presenting cells, is a weaker activator of AP-1 and CD69, allowing any incremental effect of 4-1BB signalling on CD69 expression to be detectable with SEB but not with PMA/ionomycin. One alternative explanation suggested by our signalling data is the possibility that the 4-1BB endodomain exerts an influence on early signalling events such as ZAP70, upstream of protein kinase C. As such, a stimulant that acts at the TCR, such as SEB, may have its effects enhanced by the 4-1BB endodomain compared to a stimulant that acts downstream of ZAP70, such as PMA/ionomycin. Indeed, hypo- and hypermorphic mutations in human T-cell signalling pathways are similarly distinguished by differential responses to TCR stimulation and PMA/ionomycin [52].

In contrast, CARs with a CD28 endodomain did not demonstrate any altered function compared to untransduced or Mutζ T-cells. However, the possibility that different results may occur with a CD28 transmembrane and hinge regions cannot be excluded [53]. The differences between 4-1BBζ and CD28ζ CARs may also relate to the differing biology of the costimulatory domains as noted previously [12]. Finally, in our experiments it is unlikely that the CD28ζ CARs were functionally exhausted or anergic as seen with other CD28ζ CARs [8]. Although there was higher PD1 expression in CD28ζ CARs, they were not functionally impaired compared to untransduced controls, nor was their scFv-mediated cytotoxicity impaired. Furthermore, PD-L1 expression was not measured on target cells in this study as this has been previously noted in the literature by flow cytometric assessment [54].

One technical limitation encountered was the lower transduction efficiency encountered in 4-1BBζ CAR T-cells attributed to gradual downregulation of the gamma-retrovirally-expressed 4-1BBζ CAR during the manufacturing process [21]. It was not possible to increase the transduction efficiency for the 4-1BBζ CAR by the addition of further virus or increasing viral titre. CAR downregulation has been identified previously in 4-1BBζ CAR constructs that are manufactured specifically with gamma-retroviral non-self-inactivating vectors [21]. These 4-1BBζ CARs had greater ligand-independent NF-ΚB signalling, which acted on the CAR retroviral promoter and led to greater CAR mRNA expression per viral copy number, greater apoptosis and poorer expansion when compared to CD28ζ CARs or 4-1BBζ CARs whose CAR expression was attenuated by an internal ribosome entry site. As such, a gradual decrease in CAR expression in these 4-1BBζ CARs was noted, presumably to attenuate ligand-independent signalling, or via selective deletion of high 4-1BBζ CAR-expressing cells. We chose not to reduce the transduction efficiency of the other SS1 CAR T-cell products to match the 4-1BBζ CARs, as that would not be representative of the typical manufacturing process. We also chose not to express the 4-1BBζ CAR with a separate lentiviral vector so that any comparisons could be attributed to the costimulatory domain rather than the viral vector. Instead, the flow analysis was gated on CAR+ T-cells, thus controlling for variations in transduction efficiency whilst also ensuring uniformity in the manufacturing method. However, variation in CAR expression levels may still influence the results. Importantly, sorting and enrichment of CAR T-cells was not performed to avoid any potential stimulation of the CAR via the scFv. SS1 CAR constructs were also intentionally constructed without marker protein tags so that any change in T-cell biology could be attributed to the CAR and not reporter genes. However, the reliance on protein L prevented the use of canonical TCR stimulants such as anti-CD3/CD28 antibodies. In an ideal situation, an anti-idiotype antibody could avoid these problems but unfortunately no such antibody currently exists for the SS1 scFv.

While SEB and pervandate robustly stimulate a broad range of T-cells and are commonly used in assays of T-cell function and signalling, these stimulants have limitations. Firstly, they do not assess physiological interactions of antigen-specific TCRs with antigen. The assessment of clonally selected tumour-specific or viral-specific T-cells would be an alternative and perhaps more physiological model. However, the disadvantage of using tumour-specific or viral-specific T-cells is that the population of such T-cells in peripheral blood is small, greater expansion is required to generate cell products, and this could introduce confounders such as exhaustion during in vitro manufacturing. In addition, models using antigen-specific T-cells would exclude important subsets of T-cells such antigen-naïve T-cells. Another disadvantage related to SEB, is that only a fraction of T-cells from each donor responds to SEB based on the individual’s Vβ repertoire. As this fraction can vary between donors, it can lead to variability between donors, making it difficult to exclude small differences in function between cell products, for example among the CD4+ CAR T-cell products. While SEB as a stimulant could contribute to reduced sensitivity, it also makes the statistically significant differences identified with the CD8+ 4-1BBζ CAR more noteworthy.

These results have intriguing clinical implications. If CD8+ 4-1BBζ CARS have increased functional responses to SEB, they may also have increased functional responses to neoepitopes in malignancy in epitope-spreading. In addition, given that the management of malignancy can lead to immunocompromised states, another possibility is that CD8+ 4-1BBζ CAR T-cells may be superior to the other CAR counterparts in controlling infection. Third, there is the potential downside that CD8+ 4-1BBζ CARs could lead to a greater risk of autoimmunity. While reports of autoimmunity in the context of CAR T-cell therapy are rare and causality difficult to establish [55, 56], it is possible that this may become more common given that CARs are now being considered in combined therapies with immune checkpoint blockade, and 4-1BBζ CARs have the potential to be long-lived. One further implication for CAR T-cell research is that when a toxic side-effect is noted, it is increasingly important to assess the TCR clonotype that may be involved, to ensure that the side-effects are not mediated via the TCR as opposed to the scFv. Finally, our results would cohere with the data from Ghosh et al. suggesting that 4-1BBζ CARs have a greater risk of graft versus host (GVHD) disease in allotransplantation compared to CD28ζ CARs [12]. To date, donor-derived anti-CD19 CD28ζ CAR T-cells after allogeneic haematopoietic stem cell transplant are also associated with low rates (≤ 10%) of GVHD [57–59]. Of interest, in one trial of donor-derived anti-CD19 4-1BBζ CAR T-cells, a higher rate of GVHD has been observed (10/15 patients, 66.67%) [60]. While GVHD has not been noted as a major side-effect in “off-the-shelf” TCR-knockout allogeneic CAR T-cell products, selection of knockout cells for this is stringent [61], although consideration should be given to including ‘safety switches’.

Our analysis is confined to in vitro assays and future work in vivo is required to clarify the significance of our findings. This is essential as in vitro assays are imperfect predictors of in vivo function. Furthermore, a 24-h or even 5-days proliferation assay does not adequately assess exhaustion or persistence compared to prolonged in vivo stimulation. Given that the 4-1BB costimulatory domain has a recognized role in preventing exhaustion and enhancing persistence, and given the findings of Ghosh et al. regarding the enhanced GVHD with allogeneic 4-1BBζ CAR T-cells [12], it is hypothesized that enhanced TCR-mediated function in 4-1BBζ CARs could be even further exaggerated in vivo compared to in vitro*.* While novel biology relating to the 4-1BB endodomain has been uncovered in CAR T-cells, this would likely have even more significance to other cellular therapies that rely heavily on TCR signalling such as T-cell receptor fusion constructs (TRuCs) [62], genetically modified TCR T-cells [63] and tumour-infiltrating lymphocytes (TILs). We postulate that the addition of the 4-1BB endodomain in these constructs could create a ‘second generation’ of TRuCs or TCR-modified T-cells.

## Conclusion

While CAR T-cells have become established genetically engineered therapies, it has previously been presumed that their endogenous TCR-mediated function was not altered. Herein, we show that that CD8+ anti-MSLN SS1 4-1BBζ CAR T-cells that are stimulated via their TCR have higher levels of proliferation, activation and cytokine production. This indicates that the CAR construct alters T-cell functional responses that are unrelated to the scFv–tumour interaction. There are two possible explanations for this: augmented TCR signalling by additional ZAP70/TRAF-mediated signalling that predicts enhanced functional responses, or, more likely, an altered phenotype with an enrichment of central memory cells, which can lead to enhanced function. These explanations are not mutually exclusive. Furthermore, the significance of these findings is that CD8+ 4-1BBζ CARs would have enhanced function in the process of epitope-spreading via TCR-signalling. However, one detrimental consequence could be an increased risk of developing TCR-mediated autoimmunity. These differences are an additional consideration when choosing CAR endodomains and designing novel CAR constructs. Furthermore, the 4-1BBζ endodomain has the potential to enhance other cellular therapies if incorporated in TRuCs, TCR-modified T-cells and tumour-infiltrating lymphocytes.

### Supplementary Information

**Additional file 1: Figure S1.** (A) Flow cytometry gating strategy with sequential gating on cells, singlets, live cells and CD4+ and CD8+ T-cells. (B, C) Two gating strategies employed to identify CAR T-cells based on (B) binding of biotinylated protein L to CAR scFv followed by streptavidin-APC, or (C) intracellular anti-CD3ζ APC antibody binding to the CAR endodomain. Representative flow plots of untransduced (UT) and SS1 CAR T-cells. The antibody to CD3ζ was titrated and flow cytometer voltages optimised to distinguish between CAR T-cells and untransduced T-cells. (D) CD45RO BB515 ‘fluorescence-minus-one’ (FMO) and CCR7 PE-C7 FMO are used to establish gates for naïve, central memory, effector memory and TEMRA CD4+ T-cells respectively.

**Additional file 2: Figure S2.** CAR Cytotoxicity and MSLN expression of Capan2 and AsPC1 MSLN KO. CAR T-cell cytotoxicity against (A) Capan2, and (B) AsPC1 MSLN KO at 48 h at a normalized effector to target ratio of 2:1. Experiments were performed with n = 3 independent donors; each donor is distinguished using a unique symbol. The mean and SEM are indicated. Comparisons were made between all cell products by one-way ANOVA with Tukey’s correction for multiple comparisons. *** *p* < 0.001, * *p* < 0.05 (C) Western blot validation of MSLN knockout (KO) performed in single-cell clonal populations derived from AsPC1. A control sgRNA targeting *AAVS1* was used as a control. MIA PaCa2 is the MSLN-negative control cell line. The predicted sizes of the 40 kD mature MSLN (red) and 71 kD MSLN precursor (blue) are indicated. (D-E) Flow cytometry histograms of (D) Capan2 and (E) AsPC1 MSLN KO evaluated with FAB32652P anti-MSLN antibody (purple), matched isotype control (green) or unstained (black).

**Additional file 3: Figure S3.** The change in CD69 expression after PMA/Ionomycin stimulation. (A, B) The change in CD69 expression measured by the change in MFI-PE in CD4+ (A) and CD8+ (B) CAR T-cell products after 24-h stimulation with PMA/Ionomycin. Experiments were performed with n = 3 independent donors; each donor is distinguished using a unique symbol. The mean and SEM are indicated. Comparisons were made between all cell products by one-way ANOVA with Tukey’s correction for multiple comparisons; only statistically significant differences are indicated.

## Data Availability

All data generated and/or analysed during the current study are included in this publication and its supplementary information files.

## References

[1] Gardner RA, Finney O, Annesley C (2017). Intent-to-treat leukemia remission by CD19 CAR T cells of defined formulation and dose in children and young adults. Blood.

[2] Maude SL, Laetsch TW, Buechner J (2018). Tisagenlecleucel in children and young adults with B-cell lymphoblastic leukemia. N Engl J Med.

[3] Raje N, Berdeja J, Lin Y (2019). Anti-BCMA CAR T-cell therapy bb2121 in relapsed or refractory multiple myeloma. N Engl J Med.

[4] Brentjens RJ, Santos E, Nikhamin Y (2007). Genetically targeted T cells eradicate systemic acute lymphoblastic leukemia xenografts. Clin Cancer Res.

[5] Milone MC, Fish JD, Carpenito C (2009). Chimeric receptors containing CD137 signal transduction domains mediate enhanced survival of T cells and increased antileukemic efficacy in vivo. Mol Ther.

[6] Salter AI, Ivey RG, Kennedy JJ (2018). Phosphoproteomic analysis of chimeric antigen receptor signaling reveals kinetic and quantitative differences that affect cell function. Sci Signal.

[7] Sun C, Shou P, Du H (2020). THEMIS-SHP1 recruitment by 4–1BB tunes LCK-mediated priming of chimeric antigen receptor-redirected T cells. Cancer Cell.

[8] Long AH, Haso WM, Shern JF (2015). 4–1BB costimulation ameliorates T cell exhaustion induced by tonic signaling of chimeric antigen receptors. Nat Med.

[9] Boroughs AC, Larson RC, Marjanovic ND (2020). A distinct transcriptional program in human CAR T cells bearing the 4–1BB signaling domain revealed by scRNA-Seq. Mol Ther.

[10] Sheih A, Voillet V, Hanafi LA (2020). Clonal kinetics and single-cell transcriptional profiling of CAR-T cells in patients undergoing CD19 CAR-T immunotherapy. Nat Commun.

[11] Rejeski K, Perez A, Iacoboni G (2022). The CAR-HEMATOTOX risk-stratifies patients for severe infections and disease progression after CD19 CAR-T in R/R LBCL. J Immunother Cancer.

[12] Ghosh A, Smith M, James SE (2017). Donor CD19 CAR T cells exert potent graft-versus-lymphoma activity with diminished graft-versus-host activity. Nat Med.

[13] Jacoby E, Yang Y, Qin H, Chien CD, Kochenderfer JN, Fry TJ (2016). Murine allogeneic CD19 CAR T cells harbor potent antileukemic activity but have the potential to mediate lethal GVHD. Blood.

[14] Haas AR, Tanyi JL, O'Hara MH (2019). Phase I study of lentiviral-transduced chimeric antigen receptor-modified T cells recognizing mesothelin in advanced solid cancers. Mol Ther.

[15] Beatty GL, O'Hara MH, Lacey SF (2018). Activity of mesothelin-specific chimeric antigen receptor T cells against pancreatic carcinoma metastases in a phase 1 trial. Gastroenterology.

[16] Kaeding AJ, Barwe SP, Gopalakrishnapillai A (2021). Mesothelin is a novel cell surface disease marker and potential therapeutic target in acute myeloid leukemia. Blood Adv.

[17] Gudipati V, Rydzek J, Doel-Perez I (2020). Inefficient CAR-proximal signaling blunts antigen sensitivity. Nat Immunol.

[18] Wang L, Gong W, Wang S (2019). Improvement of in vitro potency assays by a resting step for clinical-grade chimeric antigen receptor engineered T cells. Cytotherapy.

[19] Harvey CM, O’Toole KH, Allen KN, Allen KN (2018). Chapter five - crystallization of liganded phosphatases in the HAD superfamily. Methods in enzymology.

[20] Green JM, Turka LA, June CH, Thompson CB (1992). CD28 and staphylococcal enterotoxins synergize to induce MHC-independent T-cell proliferation. Cell Immunol.

[21] Gomes-Silva D, Mukherjee M, Srinivasan M (2017). Tonic 4–1BB costimulation in chimeric antigen receptors impedes T cell survival and is vector-dependent. Cell Rep.

[22] Austin JW, Lu P, Majumder P, Ahmed R, Boss JM (2014). STAT3, STAT4, NFATc1, and CTCF regulate PD-1 through multiple novel regulatory regions in murine T cells. J Immunol.

[23] Rekik R, Belhadj Hmida N, Ben Hmid A, Zamali I, Kammoun N, Ben AM (2015). PD-1 induction through TCR activation is partially regulated by endogenous TGF-beta. Cell Mol Immunol.

[24] Hastings WD, Anderson DE, Kassam N (2009). TIM-3 is expressed on activated human CD4+ T cells and regulates Th1 and Th17 cytokines. Eur J Immunol.

[25] Carpenito C, Milone MC, Hassan R (2009). Control of large, established tumor xenografts with genetically retargeted human T cells containing CD28 and CD137 domains. Proc Natl Acad Sci U S A.

[26] Guedan S, Posey AD, Shaw C (2018). Enhancing CAR T cell persistence through ICOS and 4–1BB costimulation. JCI Insight.

[27] Secrist JP, Burns LA, Karnitz L, Koretzky GA, Abraham RT (1993). Stimulatory effects of the protein tyrosine phosphatase inhibitor, pervanadate, on T-cell activation events. J Biol Chem.

[28] Firaguay G, Nunes JA (2009). Analysis of signaling events by dynamic phosphoflow cytometry. Sci Signal.

[29] Conley JM, Gallagher MP, Berg LJ (2016). T cells and gene regulation: the switching on and turning up of genes after T cell receptor stimulation in CD8 T cells. Front Immunol.

[30] Frigault MJ, Lee J, Basil MC (2015). Identification of chimeric antigen receptors that mediate constitutive or inducible proliferation of T cells. Cancer Immunol Res.

[31] Kawalekar OU, Roddy OC, Fraietta JA (2016). Distinct signaling of coreceptors regulates specific metabolism pathways and impacts memory development in CAR T cells. Immunity.

[32] Singh N, Frey NV, Engels B (2021). Antigen-independent activation enhances the efficacy of 4–1BB-costimulated CD22 CAR T cells. Nat Med.

[33] Takahashi C, Mittler RS, Vella AT (1999). Cutting edge: 4-1BB is a bona fide CD8 T cell survival signal. J Immunol.

[34] Gonda K, Watanabe M, Tada H (2017). Quantitative diagnostic imaging of cancer tissues by using phosphor-integrated dots with ultra-high brightness. Sci Rep.

[35] Tan JT, Whitmire JK, Murali-Krishna K (2000). 4-1BB costimulation is required for protective anti-viral immunity after peptide vaccination. J Immunol.

[36] Tan JT, Whitmire JK, Ahmed R, Pearson TC, Larsen CP (1999). 4-1BB ligand, a member of the TNF family, is important for the generation of antiviral CD8 T cell responses. J Immunol.

[37] Cho E, Singh R, Han C (2023). 4-1BB-4-1BBL cis-interaction contributes to the survival of self-reactive CD8+ T cell. Cell Mol Immunol.

[38] Hennecke S, Cosson P (1993). Role of transmembrane domains in assembly and intracellular transport of the CD8 molecule. J Biol Chem.

[39] Schäfer D, Henze J, Pfeifer R (2020). A novel siglec-4 derived spacer improves the functionality of CAR T cells against membrane-proximal epitopes. Front Immunol.

[40] Majzner RG, Rietberg SP, Sotillo E (2020). Tuning the antigen density requirement for CAR T-cell activity. Cancer Discov.

[41] Ozga AJ, Moalli F, Abe J (2016). pMHC affinity controls duration of CD8+ T cell-DC interactions and imprints timing of effector differentiation versus expansion. J Exp Med.

[42] Zehn D, Lee SY, Bevan MJ (2009). Complete but curtailed T-cell response to very low-affinity antigen. Nature.

[43] Knudson KM, Goplen NP, Cunningham CA, Daniels MA, Teixeiro E (2013). Low-affinity T cells are programmed to maintain normal primary responses but are impaired in their recall to low-affinity ligands. Cell Rep.

[44] Castellanos MC, Muñoz C, Montoya MC, Lara-Pezzi E, López-Cabrera M, de Landázuri MO (1997). Expression of the leukocyte early activation antigen CD69 is regulated by the transcription factor AP-1. J Immunol.

[45] Zapata JM, Perez-Chacon G, Carr-Baena P (2018). CD137 (4-1BB) signalosome: complexity is a matter of TRAFs. Front Immunol.

[46] Isakov N, Altman A (2002). Protein kinase C(theta) in T cell activation. Annu Rev Immunol.

[47] Jutz S, Leitner J, Schmetterer K (2016). Assessment of costimulation and coinhibition in a triple parameter T cell reporter line: simultaneous measurement of NF-κB, NFAT and AP-1. J Immunol Methods.

[48] Chavez-Galan L, Ruiz A, Ramón-Luing LA (2023). The SEB1741 aptamer is an efficient tool for blocking CD4+ T cell activation induced by staphylococcal enterotoxin B. Molecules.

[49] Mandala W, Harawa V, Munyenyembe A, Soko M, Longwe H (2021). Optimization of stimulation and staining conditions for intracellular cytokine staining (ICS) for determination of cytokine-producing T cells and monocytes. Curr Res Immunol.

[50] McArthur MA, Sztein MB (2013). Unexpected heterogeneity of multifunctional T cells in response to superantigen stimulation in humans. Clin Immunol.

[51] Yin Y, Mitson-Salazar A, Prussin C (2015). Detection of intracellular cytokines by flow cytometry. Curr Protoc Immunol.

[52] Chan AY, Punwani D, Kadlecek TA (2016). A novel human autoimmune syndrome caused by combined hypomorphic and activating mutations in ZAP-70. J Exp Med.

[53] Muller YD, Nguyen DP, Ferreira LMR (2021). The CD28-transmembrane domain mediates chimeric antigen receptor heterodimerization with CD28. Front Immunol.

[54] Ashizawa T, Iizuka A, Maeda C (2019). Impact of combination therapy with anti-PD-1 blockade and a STAT3 inhibitor on the tumor-infiltrating lymphocyte status. Immunol Lett.

[55] Wang LX, Chen X, Jia M, Wang S, Shen J (2018). Arthritis of large joints shown as a rare clinical feature of cytokine release syndrome after chimeric antigen receptor T cell therapy: a case report. Medicine.

[56] Chen P, Xia Y, Lei W (2022). Case report: Hashimoto’s thyroiditis after CD19 chimeric antigen receptor T-cell therapy. Front Immunol.

[57] Davila ML, Riviere I, Wang X (2014). Efficacy and toxicity management of 19–28z CAR T cell therapy in B cell acute lymphoblastic leukemia. Sci Transl Med.

[58] Brudno JN, Somerville RP, Shi V (2016). Allogeneic T cells that express an anti-CD19 chimeric antigen receptor induce remissions of B-cell malignancies that progress after allogeneic hematopoietic stem-cell transplantation without causing graft-versus-host disease. J Clin Oncol.

[59] Kebriaei P, Singh H, Huls MH (2016). Phase I trials using sleeping beauty to generate CD19-specific CAR T cells. J Clin Invest.

[60] Liu P, Liu M, Lyu C (2020). Acute graft-versus-host disease after humanized anti-CD19-CAR T therapy in relapsed B-ALL patients after allogeneic hematopoietic stem cell transplant. Front Oncol.

[61] Condomines M, Arnason J, Benjamin R, Gunset G, Plotkin J, Sadelain M (2015). Tumor-targeted human T cells expressing CD28-based chimeric antigen receptors circumvent CTLA-4 inhibition. PLoS ONE.

[62] Baeuerle PA, Ding J, Patel E (2019). Synthetic TRuC receptors engaging the complete T cell receptor for potent anti-tumor response. Nat Commun.

[63] Daniel-Meshulam I, Horovitz-Fried M, Cohen CJ (2013). Enhanced antitumor activity mediated by human 4-1BB-engineered T cells. Int J Cancer.

