# Supplementary figures and images for: The 4-1BBζ costimulatory domain in chimeric antigen receptors enhances CD8+ T-cell functionality following T-cell receptor stimulation
Source: Cancer Cell Int. 2023 Dec 18;23:327. doi: 10.1186/s12935-023-03171-7 (PMC10726568; doi:10.1186/s12935-023-03171-7)

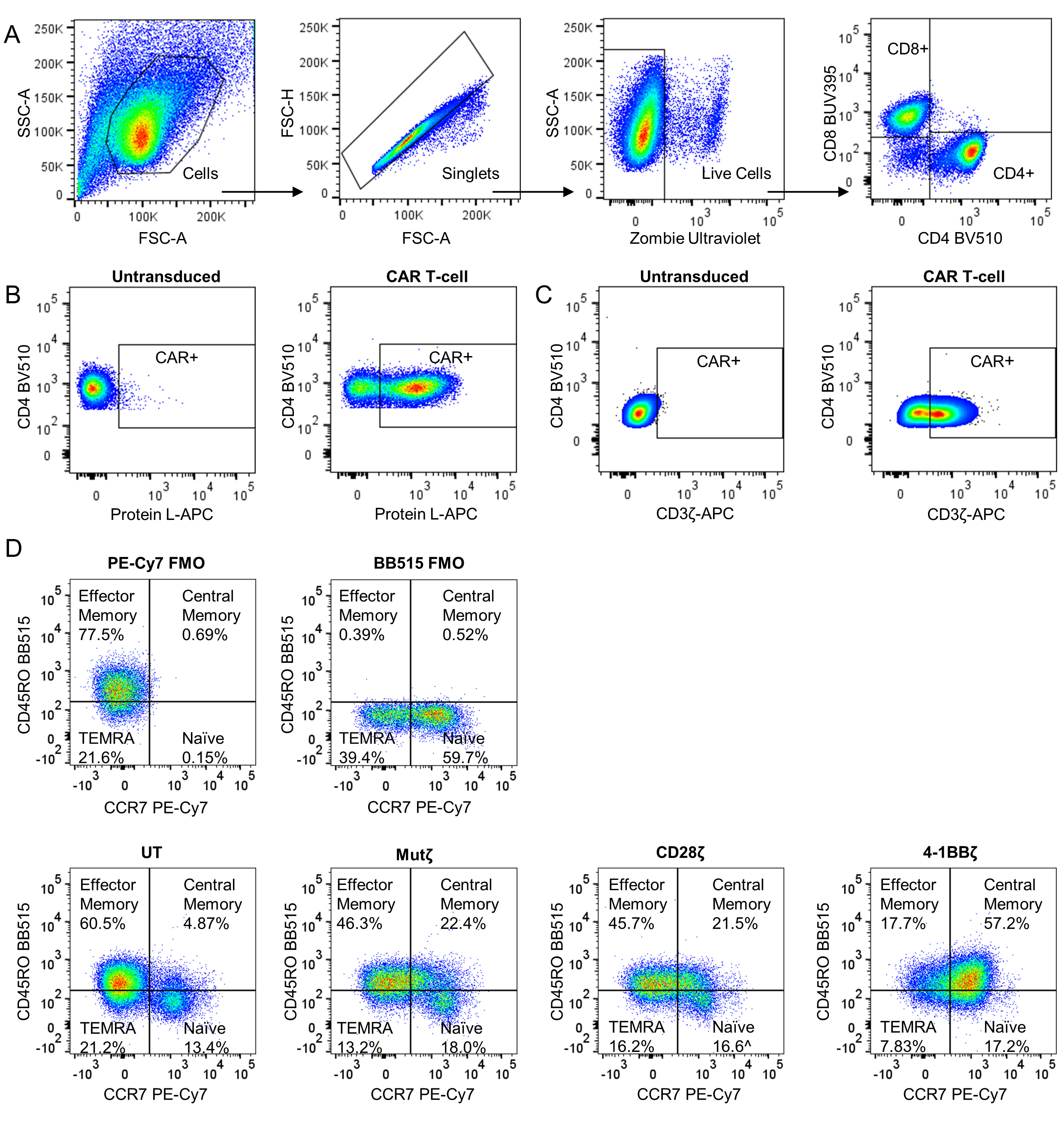

Supplement: Supplementary file 1 — Additional file 1: Figure S1. (A) Flow cytometry gating strategy with sequential gating on cells, singlets, live cells and CD4+ and CD8+ T-cells. (B, C) Two gating strategies employed to identify CAR T-cells based on (B) binding of biotinylated protein L to CAR scFv followed by streptavidin-APC, or (C) intracellular anti-CD3ζ APC antibody binding to the CAR endodomain. Representative flow plots of untransduced (UT) and SS1 CAR T-cells. The antibody to CD3ζ was titrated and flow cytometer voltages optimised to distinguish between CAR T-cells and untransduced T-cells. (D) CD45RO BB515 ‘fluorescence-minus-one’ (FMO) and CCR7 PE-C7 FMO are used to establish gates for naïve, central memory, effector memory and TEMRA CD4+ T-cells respectively. [file 12935_2023_3171_MOESM1_ESM.tif]

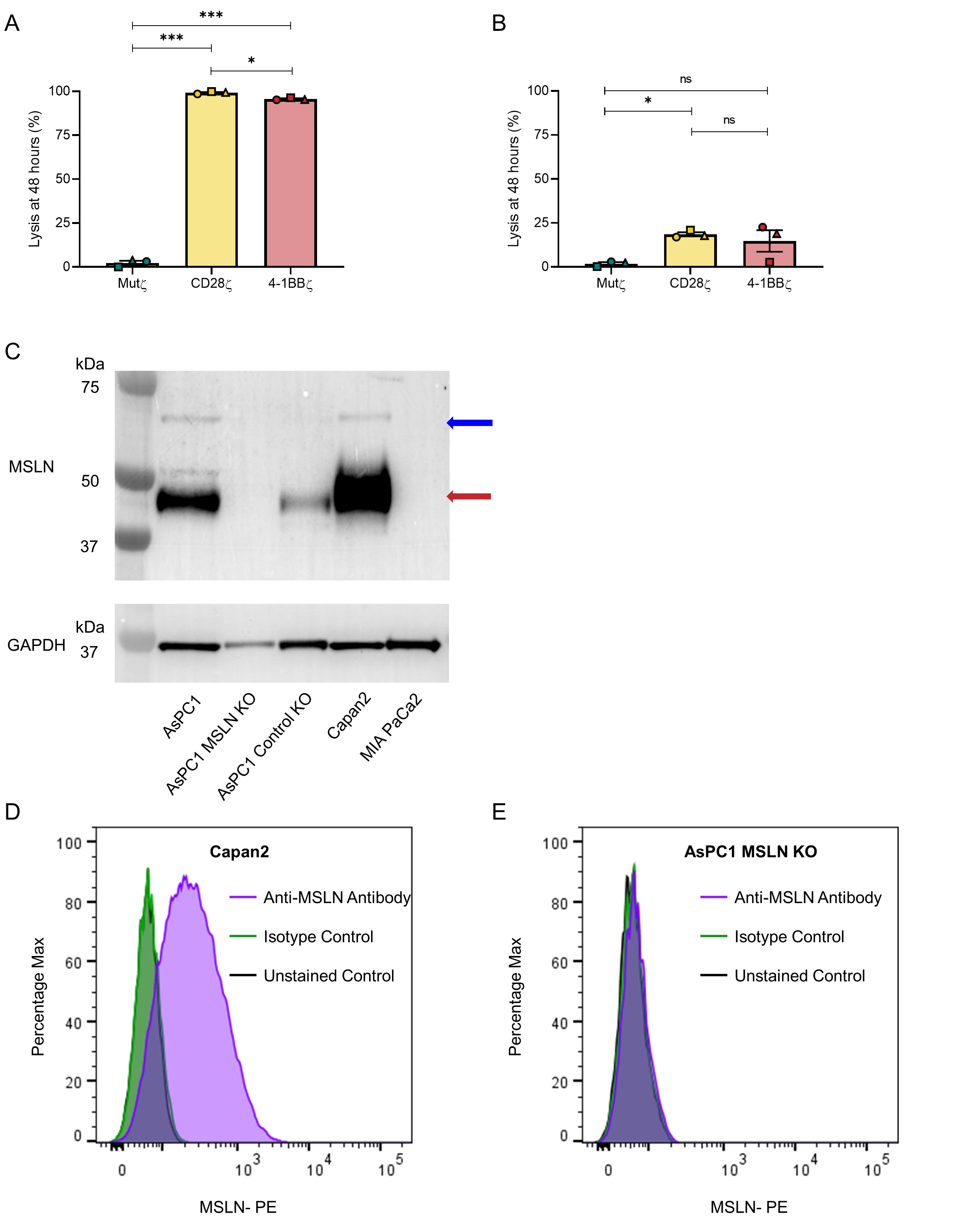

Supplement: Supplementary file 2 — Additional file 2: Figure S2. CAR Cytotoxicity and MSLN expression of Capan2 and AsPC1 MSLN KO. CAR T-cell cytotoxicity against (A) Capan2, and (B) AsPC1 MSLN KO at 48 h at a normalized effector to target ratio of 2:1. Experiments were performed with n = 3 independent donors; each donor is distinguished using a unique symbol. The mean and SEM are indicated. Comparisons were made between all cell products by one-way ANOVA with Tukey’s correction for multiple comparisons. *** p < 0.001, * p < 0.05 (C) Western blot validation of MSLN knockout (KO) performed in single-cell clonal populations derived from AsPC1. A control sgRNA targeting AAVS1 was used as a control. MIA PaCa2 is the MSLN-negative control cell line. The predicted sizes of the 40 kD mature MSLN (red) and 71 kD MSLN precursor (blue) are indicated. (D-E) Flow cytometry histograms of (D) Capan2 and (E) AsPC1 MSLN KO evaluated with FAB32652P anti-MSLN antibody (purple), matched isotype control (green) or unstained (black). [file 12935_2023_3171_MOESM2_ESM.tif]

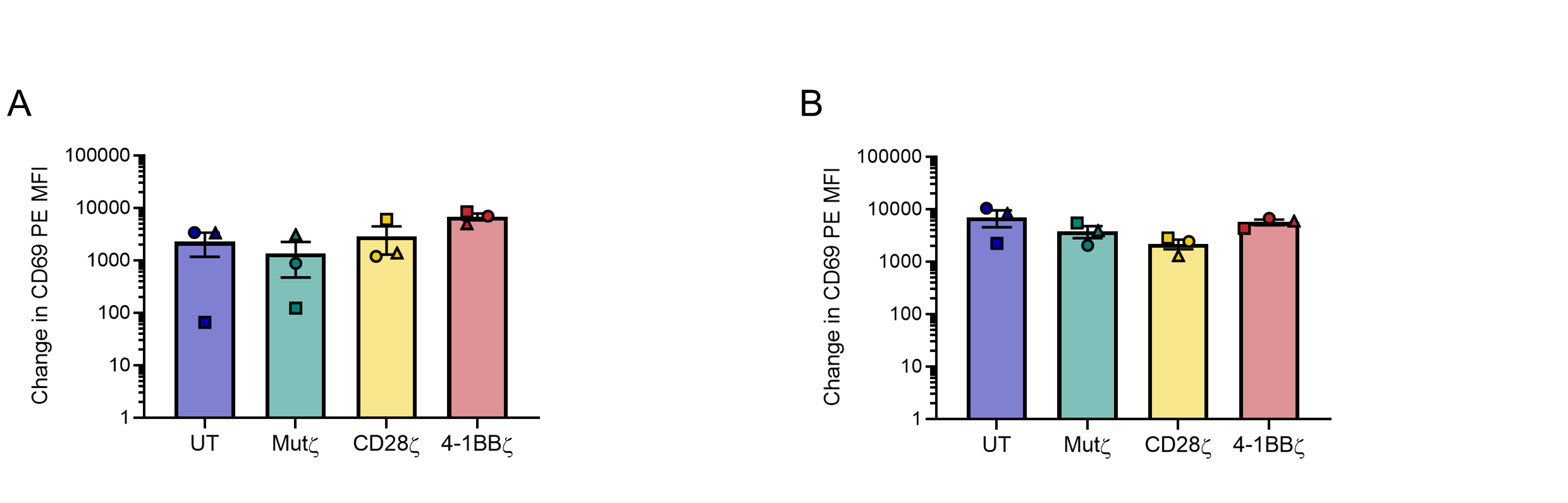

Supplement: Supplementary file 3 — Additional file 3: Figure S3. The change in CD69 expression after PMA/Ionomycin stimulation. (A, B) The change in CD69 expression measured by the change in MFI-PE in CD4+ (A) and CD8+ (B) CAR T-cell products after 24-h stimulation with PMA/Ionomycin. Experiments were performed with n = 3 independent donors; each donor is distinguished using a unique symbol. The mean and SEM are indicated. Comparisons were made between all cell products by one-way ANOVA with Tukey’s correction for multiple comparisons; only statistically significant differences are indicated. [file 12935_2023_3171_MOESM3_ESM.tif]
